# Sociodemographic and Clinical Predictors of Chronic Disease Outcomes in a Colombian Population: A Cross-Sectional Analysis of 2495 Patients

**DOI:** 10.3390/medsci14010074

**Published:** 2026-02-07

**Authors:** Adriana Guzmán Sánchez, Lilibeth Sánchez-Guette, Armando Monterrosa-Quintero, Yaneth Herazo-Beltrán, Narledis Núñez-Bravo, Carlos Andrés Collazos Morales

**Affiliations:** 1 Nursing Program, Fundación Universitaria Navarra (Uninavarra), Neiva 410001, Colombia; a.guzman@uninavarra.edu.co; 2 Facultad de Ciencias de la Salud, Universidad Simón Bolívar, Barranquilla 080001, Colombia; aliz.herazo@unisimon.edu.co (Y.H.-B.); narledis.nunez@unisimon.edu.co (N.N.-B.); 3 Ciencia y Tecnologías de la Actividad Física y el Deporte, Universidad Manuela Beltrán, Bogotá 110111, Colombia; 4 Basic Sciences and Laboratories Group, Universidad Manuela Beltrán, Bogotá 110111, Colombia; carlos.collazos@docentes.umb.edu.co; 5 Altius Performance Laboratory, Physical Education and Sports Program, Universidad Surcolombiana, Neiva 410001, Colombia

**Keywords:** non-communicable diseases, sociodemographic factors, body mass index (BMI), education, sex

## Abstract

**Objectives**: This study sought to identify sociodemographic and clinical predictors associated with the absence versus presence of alterations in mental, neurological, cardiovascular, osteomuscular, and pulmonary conditions, to provide information towards targeted interventions for non-communicable diseases (NCDs) in urban Colombian populations. **Methods**: A cross-sectional analysis was performed on 2495 patients (70.1% women) from public health facilities in Bogotá, using the Colombia Open Data “Enfermedades Crónicas” dataset collected between January and December 2023. Associations between sociodemographic variables (sex, age groups, education, and ethnicity) and clinical variables (BMI, type of disability, COVID-19 vaccination status, psychiatric risk, and the modified Medical Research Council dyspnea scale) were examined in relation to health outcomes. Data cleaning involved the exclusion of 107 outliers identified by z-scores >|3| using Microsoft Excel 365. Categorical variables were summarized using frequencies and proportions, and Pearson’s chi-square tests were applied to assess bivariate associations (e.g., BMI–health conditions, and sex–disability associations). Multivariable Firth’s penalized logistic regression models (implemented in Python 3.14 and Jamovi 2.3) were used to predict the absence of alteration (reference category: presence), adjusting for multicollinearity (variable inflation factor, VIF) and events-per-variable ratios. Odds ratios (ORs), 95% confidence intervals (CIs), and two-tailed *p*-values were estimated, with statistical significance set at *p* < 0.05. **Results**: Women predominated in obesity (81% vs. 19% in men, *p* < 0.001) and in unaltered conditions (e.g., 71% of cases without pulmonary alterations) but exhibited a lower crude prevalence of disability (6% vs. 16% in men, *p* < 0.001). Men represented higher proportions of alterations (e.g., 53.8% of pulmonary cases vs. 46.2%, *p* = 0.006) and mental disabilities (70%, *p* < 0.001). Firth regression models identified the following predictors: for mental alteration, a single COVID-19 vaccine dose (OR = 2.39, 95% CI 1.12–5.09, *p* = 0.024), occupation (OR = 1.07, 95% CI 1.05–1.10, *p* < 0.001), BMI (OR = 0.96, 95% CI 0.93–0.98, *p* < 0.001), and disability (inverted OR = 4.35, 95% CI 2.56–7.69, *p* < 0.001); for neurological alteration, occupation (OR = 1.15, 95% CI 1.10–1.21, *p* < 0.001) and disability (inverted OR = 3.45, 95% CI 1.43–8.33, *p* = 0.006); for cardiovascular alteration, BMI (OR = 1.02, 95% CI 1.00–1.03, *p* = 0.042); for osteomuscular alteration, occupation (OR = 1.03, 95% CI 1.01–1.06, *p* = 0.011); and for pulmonary alteration, occupation (OR = 1.07, 95% CI 1.03–1.11, *p* = 0.001). The models demonstrated a moderate to excellent goodness-of-fit (R^2^ = 0.25–0.72). **Conclusions**: Sex, BMI, disability status, occupation, and COVID-19 vaccination status emerged as key predictors of NCD-related alterations, highlighting specific vulnerabilities such as partial immunization for mental health risk, and disability for mental and neurological outcomes. Targeted interventions, including completion of vaccination schedules, mitigation of occupational exposure, BMI management, and disability-inclusive care, may reduce health disparities and support PAHO/WHO 2025 targets. Longitudinal studies are recommended to establish causal relationships in the context of Colombia’s fragmented subnational NCD evidence base.

## 1. Introduction

Non-communicable diseases (NCDs) represent a global public health challenge, accounting for approximately 73% of all deaths worldwide, with low- and middle-income countries (LMICs) bearing a disproportionate burden [1]. In Latin America, NCDs such as cardiovascular diseases, diabetes, chronic respiratory conditions, and mental health disorders significantly contribute to morbidity and mortality, driven by aging populations, urbanization, and lifestyle changes [2,3].

Colombia’s age-standardized mortality rate from non-communicable diseases (NCDs) currently ranges from 16.5 to 20.9 deaths per 100,000 individuals, placing the nation in the second quintile within the Americas. Over the 2010–2021 period, the rate of premature mortality attributable to the five leading NCDs declined at an average annual rate of 1.0%, which falls short of the regional interim target of –1.92% for 2025. Such a trajectory signals moderate progress but insufficient momentum to achieve the overarching objective. Meanwhile, the rise in obesity prevalence and persistent tobacco consumption amplify the overall burden of disease [4,5].

Sociodemographic variables—namely age, sex, level of education, and ethnic background— interact with clinical indicators, including body mass index and functional disability, to shape NCD outcomes; however, the synergistic effects of these determinants have yet to be rigorously examined in discrete subnational settings [6].

While the literature on non-communicable diseases (NCDs) continues to expand, critical lacunae remain regarding the interplay of sociodemographic and clinical determinants, and their capacity to forecast health trajectories across heterogeneous populations, with Colombia serving as a notable example [7]. Earlier investigations have documented correlates linking diminished socioeconomic status, constrained educational attainment, and adverse NCD trajectories; the intersection of these variables with the female sex and advanced age frequently exacerbates risk, owing to entrenched structural inequities [8,9]. For instance, a meta-analysis highlighted that a low level of education increases the risk of cardiovascular and mental health disorders by up to 37% in Latin American populations [10]. However, there is a paucity of comprehensive analyses integrating multiple predictors such as BMI, disability status, and vaccination history across various health domains (pulmonary, neurological, and mental) in Colombian settings. This is particularly relevant in the context of the COVID-19 pandemic, which has exacerbated NCD burdens due to disrupted healthcare access and increased psychiatric risks [1,2]. Moreover, publicly available datasets, such as those provided by Colombia’s Open Data portal, offer unique opportunities to explore these associations in large, representative samples, yet few studies have leveraged such resources to examine regional disparities [11].

This study aims to address existing knowledge gaps by analyzing a comprehensive dataset from the Northern Integrated Health Services Subnetwork (Subred Integrada de Servicios de Salud Norte E.S.E.), derived from the records of 2495 patients in Bogotá, to elucidate the determinants of chronic disease trajectories. The model examines a spectrum of sociodemographic covariates (i.e., sex, age, level of education, and ethnicity) alongside clinical metrics (i.e., body mass index, functional disability, and history of COVID-19 vaccination), deliberately stratifying outcomes by sex and age cohorts in light of preliminary observations suggesting disproportionate disability rates and differential engagement with health services.

Through a cross-sectional design and multivariable logistic regression methodologies, this study seeks to isolate salient and modifiable predictors capable of guiding targeted interventions and refining clinical guidelines within Colombian health systems. We hypothesize that the female sex, older age, low level of education, elevated BMI, and disability increase the odds of abnormal pulmonary, neurological, and mental health outcomes, with stronger effects in men in the pulmonary and mental domains.

## 2. Materials and Methods

### 2.1. Study Design and Data Source

This study employed a cross-sectional design to analyze sociodemographic and health-related factors associated with chronic diseases in an initial sample of 2602 participants, reduced to 2495 after the removal of outliers. The data were sourced from the “Enfermedades Crónicas” dataset, publicly available on the Colombian Open Data portal, managed under the provisions of Colombia’s Law 1712 of 2014 on Transparency and Access to Public Information.

The dataset, compiled by the Subred Integrada de Servicios de Salud Norte E.S.E., provides a comprehensive summary of chronic disease diagnoses and related clinical and sociodemographic variables. Data collection spanned from 1 January 2023 to 31 December 2023, with the dataset last updated on 17 July 2024, and metadata updated on 23 July 2024. Site: https://www.datos.gov.co/Salud-y-Protecci-n-Social/Enfermedades-Cr-nicas/2uxx-gxp3/about_data (accessed on 5 May 2025).

### 2.2. Study Population

The study population initially comprised 2602 individuals, from which 107 participants identified as outliers were excluded during data analysis (4.1%), resulting in a final sample of 2495 participants, consisting of 1749 women (70.1%) and 746 men (29.9%). The study subjects were patients who were attended at different health services within the Subred Norte E.S.E., specifically at Hospital Simón Bolívar, Hospital Engativá Calle 80, Hospital EMAUS, Hospital Suba, and the network of health centers located in Verbenal, Prado Veraniego, Bachué, San Cristóbal, Suba, Gaitana, San Luis, and Buena Vista. Eligibility for inclusion required the availability of complete sociodemographic and clinical variables documented in the institutional clinical data repository for the duration of the study. No specific exclusion criteria were applied beyond the removal of outliers, as the dataset aimed to represent the general patient population with chronic conditions.

### 2.3. Data Collection

Sociodemographic and clinical data were collected using a standardized structured form integrated into the institution’s electronic medical record system, validated and approved by the Subred Norte E.S.E. Clinical Records Committee in accordance with Colombia’s Ministry of Health Resolution 1995 of 1995. Data were gathered during initial patient interviews as part of routine clinical care. Sociodemographic variables included age, gender identity, ethnic group, educational level, and sexual orientation. Clinical variables encompassed body mass index (BMI), cardiovascular, pulmonary, neurological, mental, and musculoskeletal system status, dyspnea scale, psychiatric risk, COVID-19 vaccination status, and type of disability.

Anthropometric measurements (height and weight) were taken during medical or nursing consultations between 7:00 a.m. and 5:00 p.m. across Subred Norte facilities. Measurements were performed using calibrated equipment, including adult scales (e.g., Health o Meter models 844KL, 524KL; Seca models 813, 376; Detecto, Tanita, with capacities of 150–220 kg and a precision of 0.1 kg), pediatric scales (e.g., Health o Meter HM200P, Seca pediatric models), wall-mounted stadiometers (e.g., Charder Medical, Seca 213, range 0–220 cm, precision 1 mm), infantometers (e.g., Bioplus, Seca, precision 1 mm), and inextensible measuring tapes (e.g., Seca) for body circumferences. All instruments were regularly calibrated in accordance with the institutional maintenance and metrology program, fulfilling the specifications of Resolution 2003 of 2014.

Medical specialists, internists, cardiologists, pulmonologists, and endocrinologists, along with general practitioners, established diagnoses of chronic conditions through standardized clinical and paraclinical criteria derived from the Ministry of Health’s clinical practice guidelines. Diagnoses were recorded using the International Classification of Diseases, 10th Revision (ICD-10), with the primary diagnosis assigned based on severity and its effect on the patient’s quality of life, followed by the documentation of relevant secondary diagnoses (comorbidities); see Table 1.

The dyspnea scale was assessed using the modified Medical Research Council (mMRC) scale, internationally validated and recommended by the Colombian Association of Pulmonology and the Global Initiative for Chronic Obstructive Lung Disease (GOLD), classifying dyspnea into five grades (0–4) based on activity limitation.

### 2.4. Risk Assessments Included

- Cardiovascular risk: Evaluated using the Framingham scale adapted for Colombia (low <5%, moderate 5–9%, high ≥10% over 10 years) or the WHO cardiovascular risk scale when laboratory data (e.g., HDL, LDL, total cholesterol) were unavailable.
- Pulmonary risk: Assessed via pre- and post-bronchodilator spirometry (Spirolab III, Sipodoc), clinical evaluation, oxygen saturation, and 6 min walk test when indicated, with risk stratified using the GOLD classification (grades 1–4 based on FEV1), BODE index, and mMRC dyspnea scale.
- Neurological risk: Determined through clinical neurological evaluation, Mini-Mental State Examination (MMSE), Yesavage Geriatric Depression Scale for older adults, and vascular risk factor assessment, with severity assessed using the NIHSS (National Institutes of Health Stroke Scale) and MDS-UPDRS for Parkinson’s risk.
- Musculoskeletal risk: Evaluated clinically with joint range-of-motion measurements, Wong–Baker visual analog pain scale, numerical pain scale, Oswestry Disability Index for low back pain, WOMAC index for osteoarthritis, FRAX for fracture risk, and Downton Fall Risk Index.
- Chronic Obstructive Pulmonary Disease (COPD): Diagnosed via spirometry (FEV1/FVC post-bronchodilator < 0.7 per GOLD criteria), clinical evaluation, chest X-ray, and selective chest CT.

Disability was categorized using institutional protocols and disability certificates, following standardized criteria for cognitive, mental, motor–physical, sensory, psychological, and multiple disabilities.

### 2.5. Ethical Considerations

This study was conducted using secondary, anonymized, and publicly available data obtained from the Colombian Open Data portal (Datos Abiertos Colombia). No direct contact with participants occurred, and no identifiable personal information was accessed. Therefore, ethical review and approval, as well as informed consent, were waived in accordance with Colombia’s Resolution 8430 of 1993 for minimal-risk research, Law 1712 of 2014 on transparency and access to public information, and Law 1581 of 2012 on personal data protection. The study adhered to the principles of the Declaration of Helsinki for research involving human data.

### 2.6. Statistical Analysis

Data were analyzed using the statistical software Jamovi (version 2.3). The initial sample of 2602 participants was refined by excluding 107 outliers, yielding a final analytical sample of 2495 participants. Outliers were identified in Microsoft Excel 365 using *z*-score calculations, with values outside ±3 standard deviations considered implausible and therefore excluded.

Categorical variables were summarized using frequencies and proportions. Associations between body mass index and clustered health conditions, as well as between sex and reported disability, were examined using Pearson’s chi-square test.

Multivariate analyses were conducted using Firth’s penalized logistic regression to identify risk factors associated with mental, neurological, cardiovascular, osteomuscular, and pulmonary conditions, comparing the presence versus absence of systemic alterations. This method was selected to ensure stable parameter estimation in the presence of low-frequency outcomes. For each model, odds ratios (ORs), 95% confidence intervals (95% CIs), and two-tailed *p*-values were calculated.

Data screening and outlier identification were performed using Microsoft Excel 365. Firth’s penalized logistic regression analyses were conducted using Python (version 3.14), while complementary statistical analyses were carried out using Jamovi (version 2.3). Statistical significance was set at *p* < 0.05.

## 3. Results

This study presents a comprehensive analysis of sociodemographic and health-related factors associated with various health domains in a sample of 2495 participants.

### 3.1. Sociodemographic Characteristics

Table 2 catalogs the sociodemographic attributes of the cohort, allowing for a thorough examination of population distributions. When the sample was examined across sociodemographic dimensions, the predominance of women was evident in virtually every stratum. Among those reporting no formal education, primary, secondary, and professional qualifications, women comprised 74%, 70%, 67%, and 66%, respectively.

Sex balance approached near parity only in the technical/technological bracket, where males constituted 48% and females 52% of the cohort. The contours of gender identity revealed an overwhelming cisgender majority (99%), within which women accounted for 71% of respondents. Non-binary persons were largely men (89%), and transgender identities were nearly evenly distributed among men and women, each group constituting 50% of the small subgroup reporting such identity. For the ethnic group, most reported no particular affiliation (98%), with a similar gender distribution to the overall population, although the ROM (80%) and Raizal (89%) groups were predominantly male.

Finally, regarding sexual orientation, heterosexuality was the most frequently reported (95%), with women being the majority (70%), while other orientations, such as homosexuality (57% women) and “other” (81% women), showed a higher female proportion.

### 3.2. Health Conditions in the General Population and According to Sex

Table 3 details the frequency of selected health conditions stratified by sex, revealing pronounced disparities in outcomes between males and females. The stratification of the demographic characteristics according to sex disclosed uniform trends across the observed strata, with the female subgroup exceeding the male subgroup in nearly all classifications, as summarized in Table 2. Sociodemographic characteristics of the study population.

The analysis of the body mass index distribution disclosed a higher frequency of women than men in every quantile, with striking divergences recorded in the categories of obesity (81% of the affected subjects were females, 19% were males) and overweight (70% females, 30% males); both disproportionate distributions attained statistical significance.

In terms of pulmonary status, females showed a higher proportion in the absence of alteration category (71% vs. 29%), whereas males had a higher proportion of abnormalities (54% vs. 46%). For mental status, females also predominated in the absence of alteration category (71%), although men showed a slightly higher proportion in the presence of alteration condition (47%). Regarding psychiatric risk, men were clearly overrepresented in the low-risk (72%) and medium-risk (100%) categories, while women accounted for the majority in the no-risk group (78%). Regarding COVID-19 vaccination, females exhibited a higher dose completion, representing 76% of the cohort receiving the primary series and 68% of those achieving the booster series. Stratification by level of education revealed a consistent female predominance across all brackets, most pronounced among those without formal education (74% female) and those with only primary schooling (70%). The sex disparity diminished among individuals with technical/technological (52% female) and professional training (66% female) credentials. Finally, when analyzing the type of disability, females were more prevalent in the cognitive, motor–physical, psychological, and no-disability categories, whereas males had a higher prevalence of mental (70%) and multiple disabilities (54%).

### 3.3. Correlations Between Body Mass Index and Health Conditions

Table 4 shows the correlations between body mass index (BMI) strata and multiple health conditions, clarifying the gradient between rising BMI and increasing morbidity. As shown in Table 4. Body Mass Index vs. Various Health Conditions: The comparison of clinical conditions across the different body mass index (BMI) categories revealed statistically significant differences in all variables analyzed. Functionally, pulmonary measurements revealed that 95–97% of individuals across all BMI strata demonstrated absence of alteration; however, a 3% incidence of presence of alteration was observed exclusively within the underweight category, which was statistically significant when compared with the remainder of the sample.

With respect to neurological status, the distribution of the findings was similarly high (94–99%), yet a higher frequency of abnormal results was noted in the normal weight (2%) and overweight (1%) strata, while the underweight group presented no deviations. With respect to mental status, 91–95% of individuals were categorized as within normal limits; however, the underweight group showed the highest rate of alteration (6%), which consistently decreased across ascending BMI categories. The dyspnea characterization revealed that 96–99% of participants reported very severe symptoms, although the obesity cohort exhibited a non-negligible 4% incidence of mild dyspnea, a frequency that was statistically anomalous when compared with the remaining strata.

Finally, the psychiatric risk results showed that most individuals in all BMI groups were classified as no risk (81–89%), with a trend towards lower proportions of low risk in higher BMI categories, highest in the underweight group (19%), and lowest in the obesity group (11%). Overall, these findings suggest that underweight individuals show a higher prevalence of clinical alterations, whereas individuals with obesity tend to cluster in the normal categories, although with a higher prevalence of mild dyspnea.

**Figure 1:**
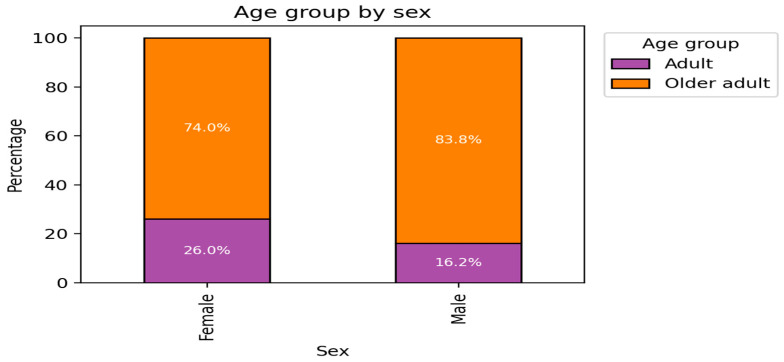
Distribution of Age Groups by Sex.

In the comparison of age distribution by sex of adults vs. older adults (Figure 1), a higher proportion of older adults was observed among males (84%) than among females (74%), whereas females had a higher proportion of adults (26%) as compared to males (16%). This difference in distribution was statistically significant, indicating that age is not independent of sex in the studied population.

**Figure 2:**
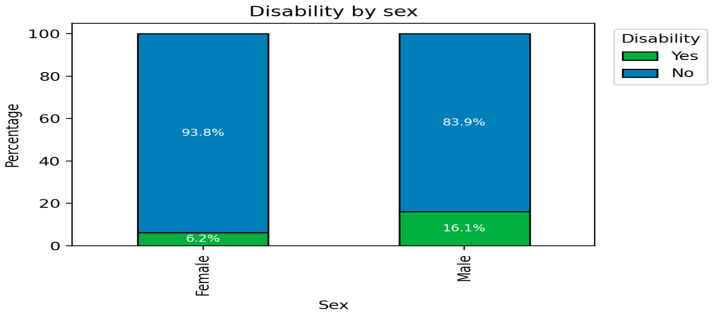
Distribution of Disability Status by Sex.

When analyzing the relationship between sex and the presence of disability (Figure 2), it was observed that disability was more prevalent in males (16%) than in females (6%), although the majority of participants in both sexes did not present any disabilities. These differences in distribution were statistically significant, indicating that disability is not independent of sex in the studied population.

### 3.4. Firth’s Penalized Likelihood Logistic Regression

Given the presence of low-frequency outcomes, Firth’s penalized logistic regression was applied to estimate the associations between potential risk factors and the presence of mental, neurological, cardiovascular, osteomuscular, and pulmonary conditions. Table 5 shows the results of the multivariate models, including odds ratios (ORs), 95% confidence intervals (95% CIs), and two-tailed *p*-values for the predictors identified across the different health domains [12].

## 4. Discussion

In this cross-sectional analysis of 2495 patients, sociodemographic and clinical variables were differentially associated with alterations in mental, neurological, cardiovascular, osteomuscular, and pulmonary systems. Women, who constituted the majority of the sample, showed a higher prevalence of obesity and a greater proportion of cases without system alterations, whereas men exhibited higher frequencies of detected alterations, particularly in pulmonary and mental domains, and a greater prevalence of mental and multiple disabilities. Body mass index, disability status, occupation, age distribution, and COVID-19 vaccination status were consistently associated with health outcomes, with the underweight status linked to higher frequencies of mental alterations and incomplete vaccination associated with increased odds of adverse mental health outcomes.

Consistent with the study hypothesis, women represented the majority of the sample (70.1%) and accounted for a larger proportion of individuals classified with obesity (81%), as well as for most cases without detected system alterations across health domains. In contrast, among participants presenting specific abnormalities, men were more frequently represented within certain affected subgroups, such as pulmonary alterations (53.8% of pulmonary abnormality cases) and mental disabilities (70% of mental disability cases). Body mass index emerged as a relevant clinical correlate: underweight individuals accounted for a higher proportion of mental alteration cases (6%) and psychiatric low-risk classifications (19%), whereas obesity was associated with a greater representation among mild dyspnea cases (4%), alongside a lower proportion of psychiatric vulnerability. These findings are interpreted as distributional patterns within clinical subgroups rather than population-level prevalence estimates [15].

The age and disability distributions revealed clear disparities: older men constituted 84% of the older-adult cohort (Figure 1), while 94% of the disability-free subgroup were women (Figure 2). These distributions reflect established trends in non-communicable diseases (NCDs) across low- and middle-income countries (LMICs), where sociodemographic inequalities magnify health burdens [8].

The application of Firth’s penalized likelihood regression (Table 5) provided a robust framework to identify critical predictors of health risks, particularly in categories with limited event frequency where standard logistic models often fail [16]. Our findings underscore a complex interplay between vaccination status, pre-existing disability, and occupational exposure. For Mental Health and Vaccination Status, one of the most salient findings was that an incomplete vaccination schedule (single dose) was associated with a 139% increase in the odds of abnormal mental risk (OR: 2.39). This association is consistent with emerging literature on post-viral neuropsychiatric sequelae [17], suggesting that partial immunization may not only fail to prevent clinical symptoms but also leave individuals vulnerable to the psychological stress of perceived susceptibility [18].

Notably, disability emerged as the most potent predictor within the mental health domain, showing a 335% increase in odds (OR: 4.34). This substantial effect is likely attributable to the heightened psychosocial barriers and isolation faced by this population [19,20]. Furthermore, disability significantly increased neurological risk odds by 245% (OR: 3.44), aligning with documented chronic neuropathic sequelae and a higher baseline vulnerability to neuro-inflammatory processes [21,22].

Occupational exposure demonstrated a consistent deleterious effect across all domains. The observed increase in neurological (+15%) and pulmonary (+7%) risks highlights the role of chronic occupational stress and environmental exposure, respectively, the latter being a known precursor to occupational stress [23,24]

In contrast, the paradoxical protective effect of BMI (OR: 0.96) in the mental health domain suggests a potential multicollinearity with body weight or specific cognitive contexts where a higher BMI might be a proxy for other unmeasured protective variables in certain populations [25].

Finally, while anthropometric variables such as height and weight reached statistical significance, their proximity to unity (OR ≈ 1.00) indicates that these results are likely a product of high statistical power rather than clinical relevance. This distinction is crucial for practical risk assessment, as it prevents the overinterpretation of negligible effects in large datasets [26].

Our sex-specific associations (Table 2), in which women predominated in obesity yet had a normal pulmonary/mental status, resonate with regional patterns; for example, dynapenic abdominal obesity in older Colombian adults was more prevalent in women, and is linked to multimorbidity via logistic regression. Similarly, BMI–health condition links (Table 3) align with studies showing an underweight status predicting psychiatric risks in urban Colombian populations, and obesity correlating with dyspnea in NCD cohorts [2].

The higher vaccination rates among women (e.g., 76% with two doses) post-COVID underscore sex disparities in health-seeking behavior, echoing PAHO reports on exacerbated NCD burdens [4,27]. These convergences validate our models’ applicability, as observed in predictive studies where sociodemographic data explained 25–35% of the variance in NCD outcomes, akin to our R^2^ values (0.25–0.72) [6].

Clinically, these findings imply the need for sex- and education-tailored NCD programs in Colombia, such as BMI-targeted interventions for underweight men to mitigate mental abnormalities, or disability-inclusive pulmonary care [28].

The public health implications include leveraging open datasets for surveillance, as disparities among ethnic minorities (e.g., 100% Afro-Colombian women, Table 1) suggest the need for culturally sensitive strategies [7]. The strengths of the study include a large, representative sample from public facilities, rigorous outlier removal, and validated tools, enhancing generalizability within urban Colombian contexts [29]. However, the limitations warrant caution: the cross-sectional design precludes causality, potentially confounding bidirectional BMI–disability links; reliance on secondary data may introduce recording biases, especially for “not recorded” categories (3–4% in systems); small subgroups (e.g., non-binary: n = 18) limit power, reflected in broad CIs; and exclusion of rural areas restricts national extrapolation, unlike broader surveys showing rural–urban diabetes gradients.

Future research should employ longitudinal designs to confirm causality, incorporate multilevel modeling for socioecological factors (e.g., structural equation modeling approaches), and expand the reach to underrepresented groups. Integrating biomarkers could refine predictors, building on our logistic framework. Ultimately, addressing these predictors could reduce Colombia’s NCD mortality, aligning with the WHO 2025 targets [1].

### Limitations

As with any cross-sectional design, our study cannot establish causality, which may overlook potential bidirectional relationships, such as abnormal health statuses potentially contributing to disability or changes in BMI over time, as suggested in similar Colombian cohorts, where reverse causation may have biased associations in NCD multimorbidity. Reliance on secondary administrative data from electronic medical records introduces potential risks of misclassification or recording biases, particularly for “not recorded” categories (3–4% across all systems), which may underestimate abnormalities if clinicians prioritized severe cases; this is a common limitation in open-data analyses, as indicated by validation studies showing 10–20% underreporting in Latin American registries. Small subgroup sizes (e.g., non-binary n = 18, ethnic minorities < 1%, abnormal outcomes < 5%) may lead to broad confidence intervals in logistic models, indicating potential instability and overfitting, especially in rare-event strata; however, the use of Firth’s penalized likelihood logistic regression in the multivariable analysis (Table 5) may mitigate this bias by providing more robust estimates and reducing perfect separation in infrequent events, as suggested in the epidemiological literature for datasets with high normality prevalence (>90% in pulmonary and neurological domains). The urban focus on Bogotá may limit generalizability to rural regions or other areas of Colombia, where NCD burdens may differ due to access disparities, as rural–urban gradients in diabetes and obesity prevalence may reach 1.5–2-fold in national surveys. Unmeasured confounders (e.g., socioeconomic status beyond education, physical activity, smoking) may explain residual variance, although our models controlled for key variables according to bibliographic guidelines. Finally, the 2023 data may precede recent post-COVID NCD surges reported in 2024–2025, potentially underestimating current psychiatric risks amid ongoing pandemics.

The overrepresentation of women (70.1%) aligns with national patterns in Colombia, where multimorbidity prevalence is higher among women (e.g., 44.2% vs. 30–35% in men, per Saavedra-Moreno et al. (2024) [30], potentially driven by greater service utilization in urban public facilities. This may inflate estimates of female-specific risks (e.g., obesity) while underestimating male vulnerabilities, such as tobacco-related pulmonary disease, which is underreported due to cultural barriers. Consequently, the findings may not fully generalize to male-dominated subgroups, risking inequitable policy recommendations that overlook men’s lower engagement with preventive care.

Furthermore, while this study leverages publicly available data to address gaps in NCD predictors within Colombian urban settings, it is constrained by the nascent state of region-specific literature in the country. Existing Colombian research on sociodemographic–clinical interactions in NCDs remains fragmented, with a heavy emphasis on national aggregates (e.g., Camacho et al., 2020 [7]) or cardiovascular/metabolic foci, often overlooking integrated analyses of pulmonary, neurological, and mental domains in subnational cohorts such as Bogotá’s public facilities. This paucity limits comparative benchmarks, particularly for ethnic minorities (e.g., ROM and Raizal groups were underrepresented here), and post-COVID psychiatric sequelae, where local studies are scarce despite rising burdens (Luciani et al., 2023 [2]). Consequently, our predictors (e.g., BMI–disability synergies) may not fully align with rural or indigenous contexts, underscoring the need for multicenter, culturally attuned longitudinal studies to refine causal pathways and to provide information towards tailored interventions under Colombia’s NCD National Plan.

## 5. Conclusions

This study identifies sex, BMI, disability, occupation, and vaccination status as robust predictors of alterations in mental, neurological, cardiovascular, osteomuscular, and pulmonary statuses in an urban Colombian population. Using open data and logistic modeling, we propose targeted interventions such as vaccination completion and occupational exposure mitigation for mental/pulmonary risks, BMI programs for underweight individuals, and educational enhancements for mental resilience to mitigate Colombia’s NCD burden, declining 1.0% annually but trailing PAHO 2025 targets. The findings support equitable policies and disability-inclusive care to reduce premature mortality. Prospective studies with diverse cohorts and advanced analytics are critical to confirm causality and scale solutions in the context of Colombia’s fragmented subnational NCD literature.

## Figures and Tables

**Table 1:** Distribution and Standardized Assessment of Chronic Disease Diagnoses in the Subred Integrada de Servicios de Salud Norte E.S.E. Dataset.

ICD-10 Category	Principal Diagnoses (Examples)	ICD-10 Code Range	*n* (%)	Normality in Related Systems
Endocrine, Nutritional, and Metabolic Diseases (E00–E89)	Diabetes, Obesity, Hyperlipidemia, Unspecified hypothyroidism	E10–E14 (Diabetes); E66 (Obesity); E78 (Hyperlipidemia); E03 (Hypothyroidism)	481 (19%)	Not directly related to pulmonary/neurological/mental/dyspnea systems. Psychiatric risk low: 100% (“No Risk” or “Low Risk”). Indirect cardiovascular link: 100% low risk.
Mental and Behavioral Disorders (F00–F99)	Unspecified anxiety disorder, Unspecified schizophrenia, Dementia, Other depressive episodes	F20–F29 (Schizophrenia); F32–F33 (Depression); F41 (Anxiety); F03 (Dementia)	48 (2%)	Mental: 88.5% “Normal”. Psychiatric risk low: 80.8% (“No High Risk…” or “Low Risk”). Neurological: 90% “Normal” (overlap with dementia). Dyspnea: N/A.
Diseases of the Nervous System (G00–G99)	Epilepsy, Unspecified migraine, Unspecified cerebrovascular disease	G40 (Epilepsy); G43 (Migraine); G45–G46 (Cerebrovascular)	25 (1%)	Neurological: 84.4% “Normal” (controlled episodes). Psychiatric risk low: 100%. Pulmonary/dyspnea: N/A.
Diseases of the Circulatory System (I00–I99)	Hypertension, Chronic peripheral venous insufficiency, Ischemic cardiomyopathy	I10 (Essential hypertension); I25 (Ischemic); I87.2 (Chronic venous insufficiency)	1434 (57%)	Cardiovascular risk: 100% “LOW RISK”. Not directly linked to pulmonary/neurological/mental/dyspnea.
Diseases of the Respiratory System (J00–J99)	COPD, Asthma, Bronchitis, Sleep apnea	J44 (COPD); J45 (Asthma); J42 (Chronic bronchitis); J47 (Bronchiectasis)	92 (4%)	Pulmonary: 94.2% “Normal” (stable cases). Dyspnea: 1.9% “Mild” (majority “VERY SEVERE”: 98%, mMRC grades 3–4). Psychiatric risk low: 100%.
Other Categories (M, N, R, Z)	Prostatic hyperplasia, Arthritis, Osteoarthritis, General medical examination, Pain	N40 (Prostatic hyperplasia); M00–M99 (Musculoskeletal, e.g., M19 Osteoarthritis); R52 (Pain); Z00 (General examination)	415 (17%)	Variable; Mental: 92% “Normal” for chronic pain. Psychiatric risk low: ~99%. Osteomuscular: High % “Normal” in joint range of motion.

Note: Data acquisition via standardized institutional history forms (Res. 1995/1999) and calibrated anthropometry (Res. 2003/2014) across sites (e.g., Hospital Simón Bolívar, CAPS Verbenal). Diagnoses confirmed by specialists using ICD-10 and par clinical criteria. Normality reflects ambulatory control (low-risk states); no “normality” descriptors in diagnostic field (all pathological). Percentages from raw counts; multiple comorbidities per patient. Statistical extensions (e.g., age-stratified risks) via software (R/Python, version 3.14) advised for full analysis. Official institutional report (Subred Norte E.S.E., Radicado 2025-009600-1, dated 21 May 2025) provides methodological validation, including informed consent under Law 1581/2012 and GOLD/mMRC scales for respiratory assessments.

**Table 2:** Sociodemographic characteristics of the study population.

Level Educational	Male	Female
No Education	207 (26%)	586 (74%)
Primary	329 (30%)	768 (70%)
Professional	10 (34%)	19 (66%)
Secondary	164 (33%)	337 (67%)
Technical/Technological	36 (48%)	39 (52%)
Total	746 (30%)	1749 (70%)
**Gender Identity**		
Cisgender	727 (29%)	1744 (71%)
Non-Binary	16 (89%)	2 (11%)
Transgender	3 (50%)	3 (50%)
Total	746 (30%)	1749 (70%)
**Ethnic Group**		
Afro-Colombian	0 (0%)	9 (100%)
Indigenous	10 (43%)	13 (57%)
None of the above	724 (30%)	1725 (70%)
ROM (Gypsy)	4 (80%)	1 (20%)
Raizal (San Andrés)	8 (89%)	1 (11%)
Total	746 (30%)	1749 (70%)
**Sexual Orientation**		
Bisexual	0 (0%)	1 (100%)
Heterosexual	719 (30%)	1663 (70%)
Homosexual	10 (43%)	13 (57%)
Other	17 (19%)	72 (81%)
Total	746 (30%)	1749 (70%)

**Table 3:** Various Health Conditions vs. Population by Sex.

BMI	Female	Male	*p*-Value
Underweight	186 (57%)	142 (43%)	<0.001
Normal weight	591 (67%)	287 (33%)	
Overweight	465 (70%)	195 (30%)	
Obesity	507 (81%)	122 (19%)	
**Cardiovascular System Status**			
Alteration Detected	12 (54.5%)	10 (45.5%)	0.109
Not Recorded	44 (62.9%)	26 (37.1%)	
No Alteration Detected	1693 (70.4%)	710 (29.6%)	
**Pulmonary System status**			
Alteration Detected	12 (46.2%)	14 (53.8%)	0.006
Not Recorded	48 (61.5%)	30 (38.5%)	
No Alteration Detected	1689 (70.6%)	702 (29.4%)	
**Neurological System Status**			
Alteration Detected	17 (51.5%)	16 (48.5%)	0.051
Not Recorded	46 (66.7%)	23 (33.3%)	
No Alteration Detected	1686 (70.5%)	707 (29.5%)	
**Mental Status**			
Alteration Detected	49 (53.3%)	43 (46.7%)	<0.001
Not Recorded	52 (59.1%)	36 (40.9%)	
No Alteration Detected	1648 (71.2%)	667 (28.8%)	
**Musculoskeletal System Status**			
Alteration Detected	46 (73%)	17 (27%)	
Not Recorded	58 (71%)	24 (29%)	0.868
No Alteration Detected	1645 (70%)	705 (30%)	
**Dyspnea scale**			
Severe	37 (82%)	8 (18%)	0.134
Moderate	21 (78%)	6 (22%)	
Light	1691 (70%)	732 (30%)	
**Psychiatric risk**			
Low risk	108 (28%)	276 (72%)	<0.001
Moderate risk	0 (0%)	2 (100%)	
No risk	1641 (78%)	468 (22%)	
**COVID-19 vaccine doses**			
Zero	154 (60%)	101 (40%)	<0.001
One	36 (64%)	20 (36%)	
Two	707 (76%)	219 (24%)	
Three	852 (68%)	406 (32%)	
**Level of Education**			
None	586 (74%)	207 (26%)	<0.001
Primary	768 (70%)	329 (30%)	
Secondary	337 (67%)	164 (33%)	
Technical/Technological	39 (52%)	36 (48%)	
Professional	19 (66%)	10 (34%)	
**Type of disability**			
Cognitive	42 (74%)	15 (26%)	<0.001
Mental	11 (30%)	26 (70%)	
Motor–physical	245 (63%)	147 (37%)	
Multiple	41 (46%)	48 (54%)	
No Present	1205 (73%)	437 (27%)	
Psychological	133 (73%)	50 (27%)	

**Note:** Statistical analysis was performed using the Chi-square (χ^2^) test to assess the association between variables; BMI = Body mass index.

**Table 4:** Body Mass Index vs. Various Health Conditions.

Pulmonary System Status				
BMI	Alteration Detected	Not Recorded	No Alteration Detected	*p*-value
Obesity	8 (1%)	19 (3%)	602 (96%)	<0.001
Overweight	1 (0.15%)	17 (3%)	642 (97%)	
Normal weight	8 (1%)	38 (4%)	832 (95%)	
Underweight	9 (3%)	4 (1%)	315 (96%)	
Total	26 (1%)	78 (3%)	2391 (96%)	
**Neurological Status**				
BMI	Alteration Detected	Not Recorded	No Alteration Detected	*p*-value
Obesity	9 (1%)	15 (2%)	605 (96%)	0.006
Overweight	5 (1%)	19 (3%)	636 (96%)	
Normal weight	19 (2%)	32 (4%)	827 (94%)	
Underweight	0 (0%)	3 (1%)	325 (99%)	
Total	33 (1%)	69 (3%)	2393 (96%)	
**Mental Status**				
BMI	Alteration Detected	Not Recorded	No Alteration Detected	*p*-value
Obesity	12 (2%)	22 (3%)	595 (95%)	0.017
Overweight	19 (3%)	19 (3%)	622 (94%)	
Normal weight	42 (5%)	35 (4%)	801 (91%)	
Underweight	19 (6%)	12 (4%)	297 (91%)	
Total	92 (4%)	88 (4%)	2315 (93%)	
**Dyspnea scale**				
BMI	Light	Moderate	Severe	*p*-value
Obesity	23 (4%)	3 (0%)	603 (96%)	<0.001
Overweight	12 (2%)	2 (0%)	646 (98%)	
Normal weight	10 (1%)	20 (2%)	848 (97%)	
Underweight	0 (0%)	2 (1%)	326 (99%)	
Total	45 (2%)	27 (1%)	2423 (97%)	
**Psychiatric risk**				
BMI	Low Risk	Moderate Risk	No Risk	*p*-value
Obesity	71 (11%)	0 (0%)	558 (89%)	0.003
Overweight	122 (18%)	0 (0%)	538 (82%)	
Normal weight	130 (15%)	2 (0%)	746 (85%)	
Underweight	61 (19%)	0 (0%)	267 (81%)	
Total	384 (15%)	2 (0%)	2109 (85%)	

**Note:** Statistical analysis was performed using the Chi-square (χ^2^) test to assess the association between variables; BMI = Body mass index; Normal = absence of alteration per clinical criteria.

**Table 5:** Penalized logistic regression analysis (Firth) for the identification of risk factors associated with mental, neurological, and systemic conditions, adjusted for multicollinearity (VIF) and events-per-variable (EPV).

Risk	Predictor	OR (95% CI)	*p*-Value	Relative Odds Increase
Mental	COVID 1	2.39 (1.12–5.09)	0.024	139%
	Occupation	1.07 (1.05–1.10)	<0.001	+7% per unit
	BMI	0.96 (0.93–0.98)	<0.001	−4% per unit
	Disability	023 (0.13–0.39)	<0.001	+335% (odds ~4.3× greater) *
Neurological	Occupation	1.15 (1.10–1.21)	<0.001	+15% per unit
	Disability	0.29 (0.12–0.70)	0.006	+245% (odds ~3.4× greater) *
Cardiovascular	BMI	1.02 (1.00–1.03)	0.042	+2% per cm
Osteomuscular	Occupation	1.03 (1.01–1.06)	0.011	+3% per unit
Pulmonary	Occupation	1.07 (1.03–1.11)	0.001	+7% per unit

**Note:** VIF: Variable Inflation Factor; BMI: Body Mass Index; * Statistical analysis was performed using Firth’s penalized likelihood logistic regression to provide robust estimates and mitigate bias from rare events [13]. For the Disability variable, the odds ratio (OR) and 95% confidence interval (CI) were mathematically inverted (relative odds increase calculated as (1/OR − 1) × 100% for OR < 1, following standard epidemiological interpretation) [14] to designate “non-disabled” as the reference group. This adjustment ensures a more intuitive clinical interpretation, representing a 335% increase in the odds of mental risk (inverted OR: 4.35, 95% CI: 2.56–7.69) and a 245% increase in neurological risk (inverted OR: 3.45, 95% CI: 1.43–8.33) for individuals with disabilities compared to those without *.

## Data Availability

The original contributions presented in this study are included in the article. Further inquiries can be directed to the corresponding authors. The data supporting the findings of this study are publicly available. The analysis was conducted using the “Enfermedades Crónicas” dataset from the Colombian Open Data portal, obtained in accordance with Colombia’s Law 1712 of 2014 on Transparency and Access to Public Information. The dataset, compiled by the Subred Integrada de Servicios de Salud Norte E.S.E., includes sociodemographic and clinical variables related to chronic disease diagnoses. Data collection covered the period from 1 January 2023 to 31 December 2023. The dataset was last updated on 17 July 2024, and its metadata were updated on 23 July 2024. The dataset is openly accessible at: https://www.datos.gov.co (accessed on 5 May 2025).

## Abbreviations

The following abbreviations are used in this manuscript:

NCDs	Non-Communicable Diseases
BMI	Body Mass Index
ICD-10	International Classification of Diseases, 10th
mMRC	Modified Medical Research Council (Dyspnea Scale)
GOLD	Global Initiative for Chronic Obstructive Lung Disease
MMSE	Mini-Mental State Examination
NIHSS	National Institutes of Health Stroke Scale
MDS-UPDRS	Movement Disorder Society-Unified Parkinson’s Disease Rating Scale
WOMAC	Western Ontario and McMaster Universities Osteoarthritis Index
FRAX	Fracture Risk Assessment Tool
COPD	Chronic Obstructive Pulmonary Disease
